# System-Level Insights into the Cellular Interactome of a Non-Model Organism: Inferring, Modelling and Analysing Functional Gene Network of Soybean (*Glycine max*)

**DOI:** 10.1371/journal.pone.0113907

**Published:** 2014-11-25

**Authors:** Yungang Xu, Maozu Guo, Quan Zou, Xiaoyan Liu, Chunyu Wang, Yang Liu

**Affiliations:** 1 School of Computer Science and Technology, Harbin Institute of Technology, Harbin, China; 2 School of Life Science and Technology, Harbin Institute of Technology, Harbin, China; 3 School of Information Science and Technology, Xiamen University, Xiamen, China; University of Missouri, United States of America

## Abstract

Cellular interactome, in which genes and/or their products interact on several levels, forming transcriptional regulatory-, protein interaction-, metabolic-, signal transduction networks, etc., has attracted decades of research focuses. However, such a specific type of network alone can hardly explain the various interactive activities among genes. These networks characterize different interaction relationships, implying their unique intrinsic properties and defects, and covering different slices of biological information. Functional gene network (FGN), a consolidated interaction network that models fuzzy and more generalized notion of gene-gene relations, have been proposed to combine heterogeneous networks with the goal of identifying functional modules supported by multiple interaction types. There are yet no successful precedents of FGNs on sparsely studied non-model organisms, such as soybean (Glycine max), due to the absence of sufficient heterogeneous interaction data. We present an alternative solution for inferring the FGNs of soybean (SoyFGNs), in a pioneering study on the soybean interactome, which is also applicable to other organisms. SoyFGNs exhibit the typical characteristics of biological networks: scale-free, small-world architecture and modularization. Verified by co-expression and KEGG pathways, SoyFGNs are more extensive and accurate than an orthology network derived from Arabidopsis. As a case study, network-guided disease-resistance gene discovery indicates that SoyFGNs can provide system-level studies on gene functions and interactions. This work suggests that inferring and modelling the interactome of a non-model plant are feasible. It will speed up the discovery and definition of the functions and interactions of other genes that control important functions, such as nitrogen fixation and protein or lipid synthesis. The efforts of the study are the basis of our further comprehensive studies on the soybean functional interactome at the genome and microRNome levels. Additionally, a web tool for information retrieval and analysis of SoyFGNs can be accessed at SoyFN: http://nclab.hit.edu.cn/SoyFN.

## Introduction

The living body is a complex system of storing and processing information. Full understanding of this system means characterising the function of its components and their interactions. The cell, as the most basic system of life, is a system of hierarchical organisation from individual molecules (such as genes, mRNAs, proteins, and metabolites) to complex molecular pathways (such as gluconeogenesis and tricarboxylic acid cycle), in which molecular interactions play an important role. Interacting molecules form functional modules (such as groups of molecules involved in the same biological process), which in turn interact with each other to drive larger scale biological processes. Comprehensive maps of the interactions among biomolecules provide an overall view of the cell. The past decade has witnessed significant effort aimed at modelling, identifying, organising, and analysing cellular interactomes. Such effort, grounded in significant advances in our understanding of molecular biology, is supported by the omic-level high-throughput data collections and acquisition techniques, which are used to interrogate the states and interactions of biomolecules at multiple levels, and to further map the structure of the genome-wide interaction networks.

**Figure 1:**
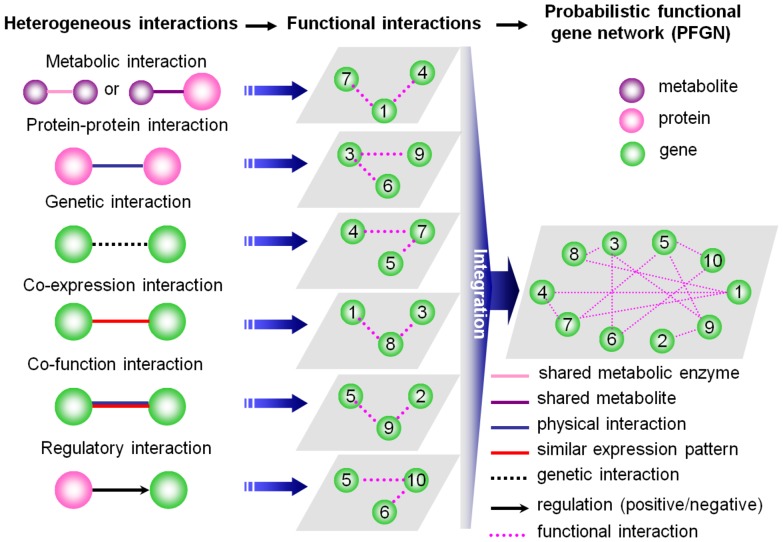
Various types of interactions between genes and a schematic view of the workflow for constructing the probabilistic functional gene networks (PFGNs).

If the complex system of a cell is regarded as a gene society, although it is in fact composed of a variety of biological molecules, the heterogeneous interactions between biological molecules are, essentially, interactions between genes. A same gene society may be modelled by various networks, of which the most popular are the protein-protein interaction network (PPIN), gene regulatory network (GRN, or transcriptional regulatory network, TRN) and metabolic network (MN). In addition, there exist various other types of connections upon which to model gene interactions, such as signal transduction pathways, co-expression networks, genetic interactions, and so forth (Figure 1). However, these models characterise the different interactive relationships between genes, implying their unique intrinsic properties and defects, and covering different slices of biological information. In other words, one specific type of connection alone cannot explain the various interactions among genes. Integrating them would contribute to a comprehensive view of the cellular system. Therefore, a challenging problem of network integration arises.

Some pioneering approaches have arisen to combine networks of different interaction types defined on the same sets of nodes, with the goal of identifying functional modules supported by multiple types of interactions. The functional gene network is such a consolidated interaction network that models fuzzy and a more generalised notion of gene-gene relations. Further, the strength of interaction between any two genes indicates the level of confidence in the functional coupling between the two genes. Insuk Lee and Edward M. Marcotte along with their colleagues [1],[2] first proposed a complete description and construction of the FGNs. They represented the specific types of interactions between genes by a more inclusive type of relations, functional interactions. The consolidation of various types of interactions with the use of the more inclusive functional interactions results in more extended coverage of genome by the gene network (Figure 1). Such consolidated interaction networks are modelled in the form of weighted graphs, where edge weights represent the likelihood of interaction between genes, estimated on the basis of various statistical models and techniques. Such a network is referred to as a probabilistic functional gene network (PFGN) [3]. So far, PFGNs have been successfully constructed for unicellular organism yeast (S. cerevisiae) [2],[4], the invertebrate nematode (*C. elegans*) [5],[6], the model plants Arabidopsis mustard (*A. thaliana*) [8],[8] and rice (*O. sativa*) [9], the mammal mouse (*M. musculus*) [10]–[12] and even the human species (H. Sapiens) [13].

Although reconstruction of FGNs, depending on a variety of function-associated data (Figure 1), has been successful in many model plant species, especially, for example, the dicot *Arabidopsis* [7] and the monocot rice [9], integrating diverse genomic data into network models for many other plants, such as soybean, is still problematic. First, the genomic data are heterogeneous in their sensitivity and specificity for relationships between genes. For example, experimental methods such as mass spectrometry preferentially observe abundant proteins, whereas comparative genomics methods apply only to evolutionarily conserved genes. Second, genomic data sets vary widely in their utility for reconstructing gene networks. Thus, we need robust benchmarking methods that can be used to evaluate each data set and allow comparison of their relative merits. Third, data sets are often correlated, but the correlations are always difficult to measure because of data incompleteness (a common problem) and sampling biases [4]. For most species, the richness and accuracy of these various function-associated data are quite inconsistent. For example, for model organisms, such as Arabidopsis, a wealth of data resources is available owing to extensive research, but for other non-model organisms, such as soybean, there are not enough data to construct such networks. We therefore need a cross-species and minimally data-dependent approach to construct the FGNs of non-model organisms.

The Gene Ontology (GO) project [14] has integrated information from multiple data sources to annotate genes to specific biological process (BP), molecular function (MF) or cellular component (CC), which are three sub-ontologies (or aspects). GO annotation (GOA) itself can be regarded as a de facto way to integrate diverse unstructured data into a single structured data source. Therefore, GOA is important for inferring FGNs based on the fact that the strength of functional interaction between genes is proportional to their functional similarity (FS). Thus we can calculate the FS among all the genes of an organism based on GOA and further construct a genome-wide network, referred to as an FGN. As a weighted network model, edge weights in the FGN represent the functional similarity rather than the likelihood of interaction between genes in a PFGN.

In comparison to the PFGN, the FGN based on GOA seems to be much easier to construct. However, construction of such a genome-wide FGN for soybean is challenging for several reasons. First, whereas *A. thaliana* has ≈27,000 protein coding genes (The Arabidopsis Information Resource, release 9) [15], soybean is predicted to have 46,430 protein coding genes, 70% more than Arabidopsis [16], but it in fact has 54174 protein-coding genes annotated by EnsemblPlants, as of May 2013 (v1.0, JGI-Glyma-1.1). This increased genome complexity results in a combinatorial explosion for the number of pairwise relations between genes (theoretically ≈1.5 billion pairs in total but actually we computed more than 2.7 billion pairs because of the three aspects of GO), complicating discovery of true functional associations. Second, the current reference knowledge and raw omic data available for modelling gene interactions are much sparser for soybean than for Arabidopsis, reducing the predictive power of resulting networks and increasing the difficulty of evaluating this power. Despite these hurdles, we constructed the first version soybean FGNs, called SoyFGNs, using the three aspects of GOA published by UniprotKB in September 2012 (version 111), which cover ≈70% of the 54174 soybean genes (Ensembls) recorded by EnsemblPlants. The construction of the second version SoyFGNs covering all 54174 genes is under way. The entire construction process described below includes the following steps: 1) measuring the pairwise functional similarities of genes annotated by GO; 2) setting a threshold to determine how similar in function the gene pairs should be to be connected in the network; 3) dissecting the validity of SoyFGNs by topology analysis, comparative analysis and functional verification.

## Material and Methods

### Datasets

#### Gene ontology (GO)

The GO data were downloaded from the Gene Ontology website [17] (data version: 1.1.3499), excluding cross-products, inter-ontology and “has-part” relationships. This dataset contains 38137 terms, including 1692 obsolete terms. The total valid terms in BP, MF and CC number 23928, 9467 and 3050, respectively. The “is-a” and “part-of” relationships number 56718 and 6127, respectively.

#### GO annotations (GOA)

The GOAs of soybean (*Glycine max*) were downloaded from UniProt-GOA (http://www.ebi.ac.uk/GOA/, version 111). A total of 165040 annotations annotate 37827 (∼70%) of the 54174 soybean genes (recorded by EnsemblPlants, release 18 April 2013). The entries annotated in BP, MF and CC number 47452, 92374 and 25214, respectively. The genes annotated in BP, MF and CC number 27594, 33189 and 14150, respectively. Here we use UniprotKB AC/IDs or Ensembl Genome IDs to represent corresponding genes.

### Functional similarities of pairwise genes

We previously proposed a shortest semantic differentiation distance (SSDD) method to calculate the semantic similarity between GO terms from a novel perspective [18]. An overlapping directed acyclic graph (DAG, a sub-graph of GO) was generated to represent two given terms. Such a DAG was then viewed as a semantic genealogy wherein a term inherits the semantics of its ancestors and distributes it to its descendants. We introduced the concept of semantic differentiation to represent the transition of a term from one pattern of semantic integration to another and the concept of semantic totipotency to represent the capacity of this differentiation. Taking into account all paths linking a term and its ancestors, the semantic totipotency of a given term 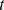 is quantified as a T-value (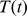) as follows:
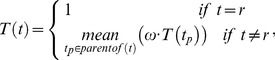
(1)where 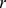 represents a root term. The semantic totipotency of the three root terms is given as 1. The variable 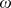 is the semantic differentiation factor for edge linking term 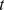 with its parent 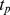. The T-values of any other terms are derived as the average of all of its parents' T-values multiplied by the semantic differentiation factor (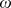). The differentiation capacity (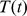) should decrease moving down the hierarchy and be positively proportional to the number of descendants, or local density. Thus, the 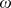 between a term 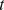 and its parent 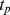 should be greater than 0 and less than 1, and can be calculated as
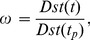
(2)where 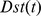 is the number of descendants of the term 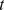, including itself.

Based on T-values, we proposed the SSDD to measure the semantic similarity in the GO. Given two terms 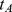 and 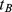, the normalised distance between them is defined as
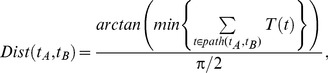
(3)where 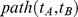 represents a set of terms on the shortest path connecting the terms 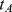 and 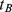 via their lowest common ancestors(LCAs). The arctan function is used to normalise the distance to (0, 1). Apparently, 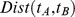 is symmetric, i.e. 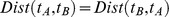. After normalisation, the semantic similarity is defined as:
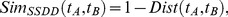
(4)


SSDD was shown to be effective for measuring the semantic similarity of pairwise GO terms. We also need a method for integrating pairwise semantic similarities into a single FS of genes because a gene is often annotated by more than one term in GOA. Three distinct approaches have been proposed for this integration: Lord et al. [19],[20] used an arithmetic average (Avg) of pairwise similarities between all terms of the first protein set and the second one; Sevilla et al. [21] used only the maximum (Max) similarity between all term pairs; Couto et al. [22], Schlicker et al. [23] and Azuaje et al. [24] developed the best-match average (BMA) method, in which each term of the first protein is paired only with the most similar term of the second one and vice versa. We take the BMA approach to compare gene similarities, as it was found to be most effective [25]. Given two genes, 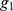 and 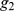, BMA is defined as
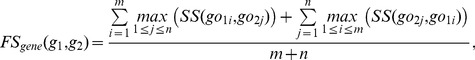
(5)


where 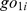 (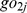) denotes a term that belongs to the term set with a size of m(n) that annotates 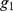 (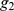). Thus, each gene pair is assigned three FSs based on three orthogonal aspects of GO. We also need a single integrated FS for each gene pair (denoted by 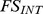). Thus, we calculate the weighted average of the three FSs as their integration (hereinafter denoted by INT), which can be formulated as
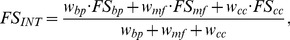
(6)where, 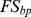, 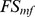, and 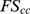 are three FSs for each gene pair; 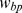, 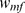 and 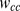 are the corresponding weights of the three GO aspects. Though the absence of a criterion to quantify the weights of the different aspects of GO on gene's function, we let the weight be equal to the corresponding FS, based mainly two considerations. First, because genes function unequally in the three GO aspects, the one yielding greater similarity should have a greater weight. Second, a great reduction in the integrated FS can be avoided even though the gene pair receives a zero FS in some aspect. The final formula for the integrated FS is
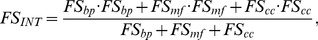
(7)where, 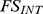 also ranges between 0 and 1.

### SoyFGNs construction

As shown in our previous work [18], our method yields more reliable gene FS for such species that has shallow gene annotations as soybean, somewhat resolving a critical problem in functional network construction. In doing so, we can calculate any pairwise FSs for a list of genes 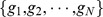, and further get an 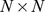 similarity matrix 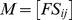, in which the element 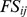 represent the functional similarity of the gene 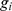 and 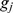. The next is to filter the matrix *M* to derive an adjacency matrix 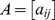 representing the functional gene network. The key to do this is to determine how similar in function must the two genes be to be linked in the network, i.e. appropriate threshold is needed to ensure that gene pairs with FSs greater than or equal to the threshold value will be connected by edges (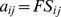); otherwise, they are not connected directly(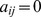).

In this study, we adopted clustering coefficient-based threshold selection. The clustering coefficient (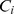) of a node (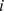) in a network is defined as 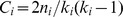, where 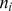 represents the number of edges between 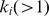 first neighbours of a gene 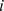; if 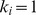, we define 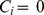. The clustering coefficient of a network is defined as the average clustering coefficient of all of its nodes,
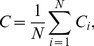
(8)


where 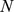 is the number of nodes in the network. If 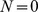, we define 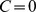.

The construction of a gene network can be viewed as a process in which links are removed from the initially complete graph by gradually increasing the FS threshold. Because all FSs range between 0 and 1, we set a series of incremental thresholds 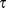 (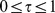) with an increment of 0.01. For each threshold 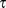, we construct a network by set 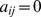 if 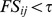. In systems biology, a genuine biological network should be scale-free and highly modular; its clustering coefficient, denoted by 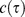, should be significantly higher than that of the corresponding random network, denoted by 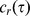. Here, we denote the difference between 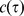 and 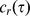 by 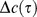, i.e. 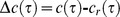. We conjectured that the most appropriate threshold should be the maximum 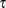, which can produce a monotonically increasing 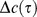 when the links are removed gradually as the threshold increases from 0 to 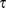. More specifically, we formulated this as a discrete optimisation problem, where the critical cut-off threshold 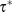 was determined by finding the first 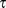, which lets 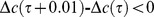 over a set of 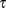 gradually increasing from 0 to 1. Note that calculating 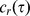 of the randomise networks is nontrivial by formula (8) because it is not clear which random network model should be used for this purpose. Hence, we adopted a statistical method proposed by Elo et al. [26] for its solution. If 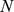 denotes the total number of nodes and 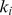 denotes the degree of a node 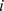 for the original network, then 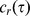 is calculated as the expected value of the clustering coefficient as follows:
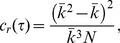
(9)


where 
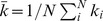
, and 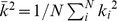.

Finally, an FGN can be constructed and represented as 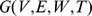, where 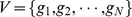 represents the genes involved in the network, 
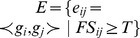
 represents the edges between gene pairs with FSs greater than or equal to the threshold *T*, 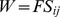 represents the weights of the edges, which are the FSs of pairwise genes.

Using the pairwise FS of all soybean genes and the clustering coefficient-based threshold selection, we construct four soybean functional gene networks (SoyFGNs) in BP, MF, CC and INT, respectively.

### Topologic characterisation of SoyFGNs

One way to characterise biological networks is to study their topologic properties. We using Cytoscape 2.8.2 [27], investigated the global properties of the resulting SoyFGNs. In addition, we conducted an in-depth analysis of the degree distribution and degree correlation, as described in the next two subsections.

#### Degree distribution

Many early studies observed that biological networks are generally scale free and their degree distribution follows the power law [28],[29]. A number of later studies have argued that there are other distributions, such as the log-normal distribution, which explain the degree distribution better than power law [30],[31]. We used three models to investigate the distributions of the four resultant FGNs: lognormal, power law and exponential. All model fittings and visualisations are completed with the use of Origin 9 (http://www.originlab.com).

#### Degree correlation

Degree correlation is a basic structural metric for calculating the likelihood that nodes link to nodes of similar or dissimilar nodal degree. The former case is called positive degree correlation, and the latter is called negative degree correlation. In the social sciences, a network with positive degree correlation is referred to as an assortative network, whereas a network with negative degree correlation is referred to as disassortative network [32]. Three ways of characterising the amount of degree correlation are used, each involving less detail and expressing the result in more compact terms. They are the joint degree distribution (JDD), the k-nearest neighbours (*knn*) and the Pearson degree correlation (PDC).

The JDD is defined as the distribution in which each entry 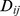 is the number of edges that the nodes at their endpoints have degrees *i* and *j*, respectively. JDD is actually a two-dimensional distribution of the number of edges with respect to the degree of their connected nodes.

Instead of recording every pair of nodes, as JDD does, *knn* simply averages the degrees of the neighbours of each node of a given degree and plots the results as linear, semi-log, and log-log plots. If a degree is missing, it is skipped in the graph. A rise in *knn* along with a rise in nodal degree indicates that nodes of similar degree tend to be linked, whereas a fall in *knn* with a rise in degree indicates the opposite.

PDC is the most condensed way to characterise the degree-link structure of a network. It consists of the conventional Pearson correlation calculation applied to each pair of linked nodes. The result always lies in the range [−1, 1], with a negative value indicating that nodes of dissimilar degree tend to be linked and a positive value indicating that nodes of similar degree tend to be linked.

### Evaluating SoyFGNs through comparison to a network generated by orthology from Arabidopsis

#### A Soybean network generated by orthology from Arabidopsis

An alternative approach to constructing a soybean gene network might be simple to transfer linkages from orthologous gene pairs of the existing gene network. This approach does not require modelling using soybean annotations or any of the soybean-derived experimental data. The value of this approach has been shown in reconstruction of gene networks for *C. elegans* [5] and *Arabidopsis* [7]. To assess the accuracy of the SoyFGNs in comparison to such an orthology-derived network, we first identified the orthologs between soybean and Arabidopsis using BLASTN, the results are shown in Table S1. We then downloaded the gene network of Arabidopsis from BioGRID (3.2.96) and infer soybean gene linkages based on linkages of this network, generating an orthology-derived soybean gene network, which consists of 16566 nodes (genes) and 146562 edges (linkages).

#### Inferring functional linkages from KEGG pathways and validating a query network

To validate SoyFGNs using independent annotations, we employed the Kyoto Encyclopedia of Genes and Genomes (KEGG) pathway database [33]. KEGG is based on manual curation and is thus considered generally accurate and largely independent from both SoyFGNs and the orthology-derived network. We downloaded equivalent link information for soybean genes from LinkDB (http://www.genome.jp/linkdb/) using UniprotKB AC/ID on March 2013. All links were also mapped to Ensembl Genomes IDs. As a result, 3145 genes were mapped to 238 pathways, which can be retrieved by our web database (http://nclab.hit.edu.cn/SoyFN/tar_pathway.php). As a benchmark network, the KEGG-derived network was constructed by generating linkages between genes sharing KEGG annotation terms, i.e. sharing the same KO IDs. The validation of a query network by KEGG-derived network is mainly based on the gene coverage and the linkage accuracy. The gene coverage (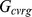) is defined as 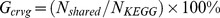, where 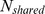 is the number of genes shared by the query network and the KEGG-derived network, 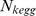 is the number of genes involved in KEGG-derived network. The linkage accuracy (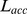) is defined as 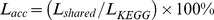, where 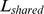 is the number of linkages between 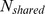 genes in the query network, 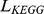 is the number of linkages between 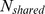 genes in the KEGG-derived network.

#### Inferring functional linkages from co-expression data and validating a query network

Another major source of functional associations is mRNA co-expression data. So we additionally inferred functional associations from mRNA co-expression profiles to evaluate SoyFGNs. 11 datasets for *Glycine max* genes was downloaded from the Gene Expression Omnibus (GEO) [34] on March 2013 (Table 1). In order to reduce the false positive rate, 4 datasets that have less than 20 samples each were discarded. The remaining 7 datasets were then filtered by removing the uninformative sets by testing for a significant correlation between the Pearson correlation coefficients (PCCs) between pairs of genes' expression vectors and removing the genes not sharing a specific Ensembl Genomes ID for further analysis. For each dataset, the PCC between pairs of genes' expression profile was used as the measure for inferring the co-expression linkages. The pairs of genes, between which the absolute value of PCC is more than 0.8, were linked. Finally, all linkages derived from 7 expression datasets were merged into a final co-expression network. The inclusiveness of a network versus co-expression network is also measured by the gene coverage (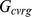) and the linkage accuracy (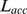).The gene coverage (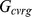) is defined as 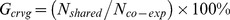, where 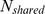 is the number of genes shared by the query network and the co-expression network, 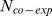 is the number of genes involved in co-expression network. The linkage accuracy (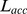) is defined as 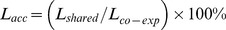, where 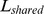 is the number of linkages between 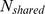 genes in the query network, 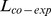 is the number of linkages between 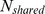 genes in the co-expression network.

**Table 1:** Soybean mRNA expression datasets and the inferred functional linkages.

Accession	Series	Title	# samples	# genes	# inferred linkages
GDS3229	GSE9374	Transgenic and conventional cultivar comparison	25	9971	1810344
GDS3230	GSE8432	Fungal pathogen Phakopsora pachyrhizi effect on leaves: time course	25	12723	3639596
GDS3231	GSE9730	Lipochitooligosaccharide effect on first trifoliolate leaf	6	-	-
GDS3234	GSE7108	Leaf response to fungal pathogen Phakopsora pachyrhizi	6	-	-
GDS3235	GSE8112	Early maturation-stage seed compartments	34	4911	88776
GDS3238	GSE6414	Globular-stage seed compartments	28	7271	183976
GDS3239	GSE7511	Heart-stage seed compartments	23	10067	407522
GDS3240	GSE7592	Scarlet Runner Bean globular-stage embryo	4	-	-
GDS3241	GSE7881	Cotyledon-stage seed compartments	18	-	-
GDS3242	GSE7124	Effect of host quantitative resistance during Phytophthora sojae infection: time course	128	6553	1344441
GDS3244	GSE9687	Phytophthora sojae infection effect on hypocotyl sections: time course	160	6756	367047
Merged co-expression network				12933	2971228

Accession numbers reference the GEO datasets. The dashes (-) represent the discarded datasets that have less than 20 samples each.

For the reason that co-expression network is generated from an approach different from the one to generate GO, while the KEGG network is generated from the same approach to generate GO. It would generate a very low linkage accuracy. To evaluate the perhaps low linkage accuracies are statistically significantly higher than the background accuracy, we made an additional statistical analysis between the linkage accuracies of the original ontology-derived network and SoyFGNs and their corresponding randomized networks. A randomized network is generated by doing randomly perturbations to the edges, but maintaining the same nodes and their degree distributions. As our pre-experiments showed that more than 400 times perturbations could provide a stable-property randomized networks, we used the average of 400 randomized networks to evaluate the background linkage accuracy. The p-values are given to indicate their difference significances (by ANOVA).

### Network-guided disease-resistant gene discovery

The aforementioned pathway and co-expression analysis showed that genes for similar biological processes or with similar expression profiles can be successfully associated in SoyFGNs. We next, as a case study, specifically tested the feasibility of predicting the genes governing plant disease resistance by using SoyFGN-INT in two steps: network-guided discovery and in silico verification.

Plant disease resistance protects plants from pathogens. Resistance genes (R-genes) are genes in plant genomes that convey plant disease resistance against pathogens by producing R-proteins. The main classes of R-genes consists of a nucleotide binding domain (NB) and a leucine rich repeat (LRR) domain(s) and are often referred to as (NB-LRR) R-genes. NB-LRR R-genes can be further subdivided into toll interleukin 1 receptor (TIR-NB-LRR) and coiled-coil (CC-NB-LRR) [35]. To implement this study, randomly selected 24 genes were used as query genes to predict R-genes using the Gaussian smoothing guilt-by-association method [36]. In order to evaluate the stability of prediction, 6 (1/4) of 24 query genes were putative R-genes, while others were experimentally verified R-genes (see Table S2). To be noted that, we here predicted candidate genes by only using the direct network neighbors via guilt-by-association. 225 of 737 candidate genes, which are highly connected with 14 query R-genes and constitute the biggest disease resistant module, were used for further analysis (see Table S3). For these 225 disease-resistant candidates, we first defined their functions by extensive databases and literatures searches. Second, we assigned a weighted rating (WR) score for each candidate according to its connected known R-genes to prioritize their possibilities to be R-genes. Obviously, WR of a gene should be positive proportional to both the number of its neighbor function-known R-genes and the average weight of edges link it to the neighbors, which were represented as functional similarity (FS). We used the so called ‘true Bayesian estimate' to compute such *WR*, which is a useful weighting mechanism used by the Internet Movie Database (IMDb) to adjust a movie's rating score based on the number of votes it has received. The formula is defined as:
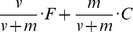
where *F*, the average FS of each gene; *C*, the total average FS of all genes; *v*, the number of neighbor function-known R-genes; *m*, a minimum number of neighbors to a R gene, which was set to be the first quartile of the neighbor number distribution of all 225 genes. The resulting *WR* scores of 225 genes are also provided in Table S3 (xls).

## Results

### Functional similarities of pairwise genes

**Figure 2:**
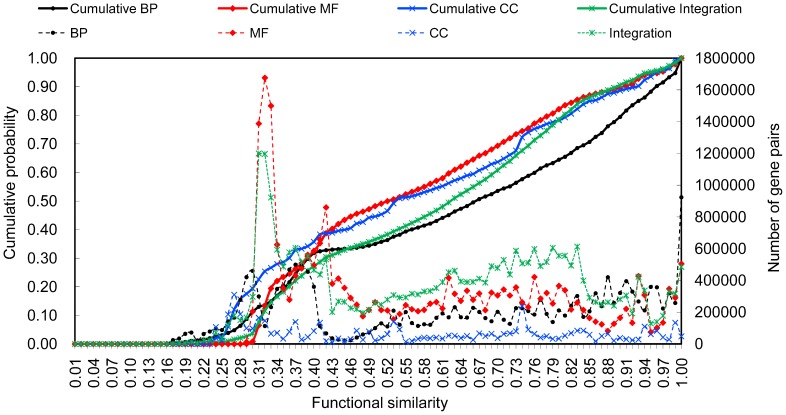
F**igure 2.** The distribution of pairwise functional similarities of soybean genes (dashed lines with marks) and the cumulative probabilities of distributions (solid lines with marks).

Measuring the pairwise FSs of soybean genes is the first step of SoyFGNs construction. UniProt-GOA (http://www.ebi.ac.uk/GOA/), published in September 2012 (version 111), deposit 165,040 annotations, annotating 37,827 (∼70%) of the 54174 soybean genes in total (recorded by EnsemblPlants, release 18-April 2013). The numbers of genes annotated by BP, MF and CC are 27594, 33189 and 14150, respectively. Using our previously proposed SSDD [18], we obtained 380700621 (27594*27593/2), 550738266 (33189*33188/2), and 100104175 (14150*14149) pairwise FSs in BP, MF, and CC respectively. We then assigned each gene pair with an integrated FS using the weighted average of three FSs (see METHODS for details), producing 715422051 (37827*37826/2) pairwise FSs of 37827 genes, referred to as “Integration (INT)”. We excluded the FSs of the genes themselves because these will not be used for subsequent construction of the no-loop networks. The distribution of these four types of pairwise FSs is shown in Figure 2. The complete data are provided on our website (http://nclab.hit.edu.cn/SoyFN) because their sizes exceed the upper limit of supplementary files (each larger than 8 GB). All genes can be retrieved by the UniprotKB AC/ID or the Ensembl Genome ID on our website (e.g., K7MVA4 and GLYMA18G52145). Hereafter in this paper, we use the UniprotKB AC/ID to refer to the corresponding gene.

### SoyFGNs construction

Our SoyFGNs are weighted undirected graphs in which the nodes represent genes and the edges represent their functional associations weighted by the pairwise functional similarities (FSs) of genes they link. Given pairwise FSs, the next step is to set an appropriate threshold to ensure that gene pairs with FSs greater than or equal to the threshold will be connected by edges; otherwise, they are not connected directly. We adopted the clustering coefficient-based threshold selection, which is based on the fact that given a threshold 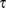, a biological network should be scale-free and highly modular, and thus its average clustering coefficient, denoted by 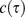, should be significantly higher than that of the corresponding random network, denoted by 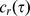.

**Figure 3:**
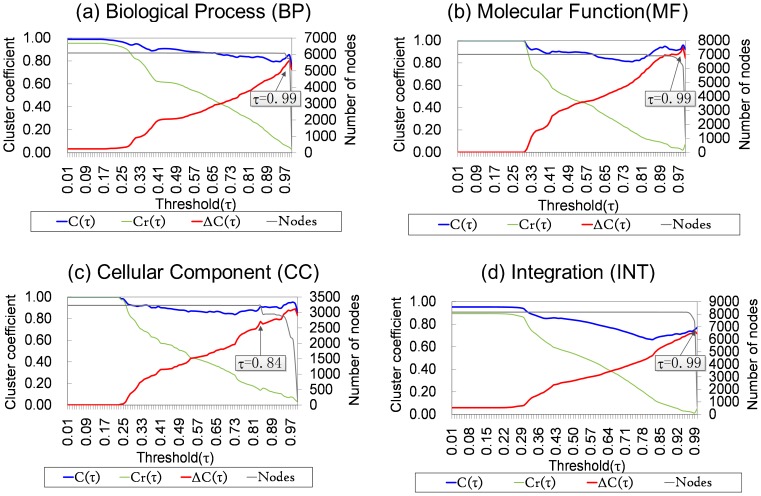
Cluster coefficient and nodes number of the network under each threshold in BP, MF, CC, and Integration (INT), respectively. Black arrows point to the first peaks of the red curve and rectangular boxes show the corresponding threshold values. 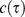 represents the cluster coefficient of the created network (blue curve), 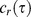 the cluster coefficient of the corresponding random network (green curve) and 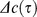 the difference between 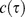 and 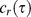 (red curve). “Nodes” represents the number of nodes at each threshold (grey curve).

By setting a series of incremental thresholds 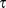 (from 0 to 1)with an increment of 0.01, we used each threshold to filter the original networks (including all pairwise similarities of genes) in BP, MF, CC and INT, respectively. As a result, we obtained 100 networks each in BP, MF, CC and INT. Using our own JAVA script, we calculated the cluster coefficient of each network (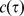) and that of its random model (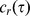). As shown in Figure 3, the first stop of monotonically increasing of the 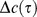 occurs at 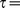 0.99, 0.99, 0.84, and 0.99 in BP, MF, CC, and INT, respectively, which indicates that these thresholds are the most appropriate ones for constructing the FGNs in BP, MF, CC, and INT, respectively (for more explanation, see the corresponding parts of the METHODS section).

Using the above-mentioned thresholds, we constructed four FGNs in BP (SoyFGN-BP), MF (SoyFGN-MF), CC (SoyFGN-CC), and INT (SoyFGN-INT) (Table 2).

**Table 2:** Summary properties of soybean functional gene networks (SoyFGNs) in BP, MF, CC, and INT.

Property	SoyFGN-BP	SoyFGN-MF	SoyFGN-CC	SoyFGN-INT
Number of nodes	25835	28833	14136	33807
Number of edges	7366700	8552866	6144656	9187249
Cluster coefficient	0.8521	0.9594	0.9107	0.7522
Connected components	137	119	76	38
Diameter	11	9	11	10
Radius	1	1	1	1
Centralisation	0.04196	0.07967	0.09372	0.1325
Shortest paths	9337684	1982914	1739716	51359370
Characteristic path length	3.366	2.0965	3.90382	3.7753
Avg. number of neighbors	139.7488	166.0099	128.96774	132.9974
Density	0.025	0.0273	0.0482	0.0182
Heterogeneity	0.8835	0.9205	0.8464	1.0899

All properties are calculated by Cytoscape 2.8. The suffix BP refers to biological progress; MF, molecular function; CC, cellular component; INT, integrated network based on the integrated functional similarity.

### Topologic characterisation of SoyFGNs

#### Global topologic properties of SoyFGNs

Analysed by Cytoscape 2.8.2, the global properties of the functional gene networks in BP, MF, CC, and INT are shown in Table 2. These four networks cover 25835 (93.63% of 27594), 28833 (86.88% of 33189), 14136 (99.90% of 14150) and 33807 (89.37% of 37827) genes (recorded by UniprotKB-GOA, version 111 September 2012) of Soybean, respectively. All networks manifest the typical common characteristics of biological networks: high clustering coefficient, small diameter and low density, and high centralisation.

#### Degree distribution

**Figure 4:**
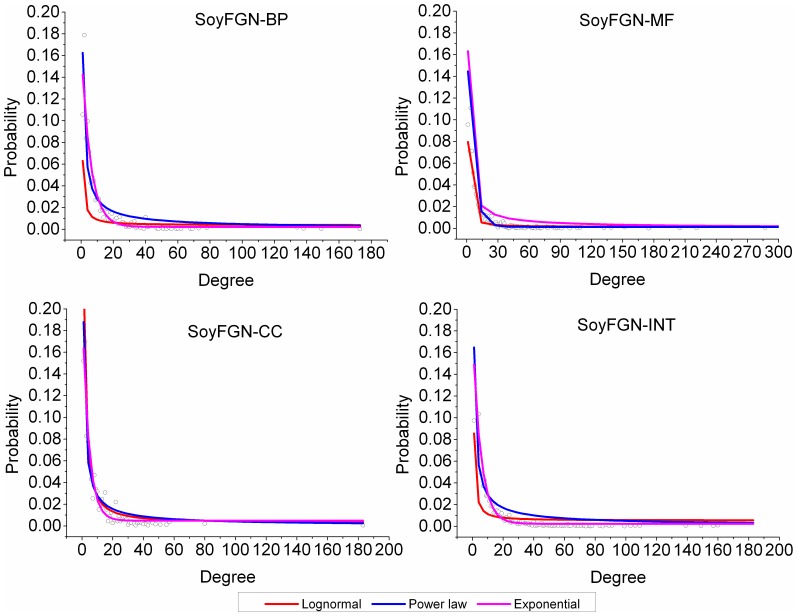
The graphic view of the degree distributions and fitted models for each functional gene network.

Three models were used to investigate the distributions of the four SoyFGNs: lognormal, power law, and exponential. Graphs of the degree distribution and the three fitted models for each network are shown in Figure 4. The detailed parameters of these models and their performances (represented by R-squared, R^2^) are listed in Table 3. Our results showed that the exponential models followed by power law models fit the degree distribution best, and the lognormal models were the worst. The degree distributions indicate that SoyFGNs have the typical characteristics of biological networks, e.g., scale free, small world, rather than the characteristics of random networks, for which the degree distribution fit Poisson distribution best. We would like to clarify that Poisson distribution was also used to fit the degree distribution of SoyFGNs, but the results are not given because they deviated fully from the degree distribution of each network.

**Table 3:** Three types of fitted models of the degree distribution for each network.

Model	Parameter		SoyFGN-BP	SoyFGN-MF	SoyFGN-CC	SoyFGN-INT
Lognormal 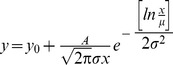		*y_0_*	0.00±0.012	0.00±0.007	0.00±0.006	0.01±0.017
		*µ*	0.14±34.14	0.37±15.400	3.69±3.901	0.25±20.765
		*σ*	6.82±325.768	4.32±51.178	2.40±1.723	4.23±90.514
		*A*	1.06±7.539	0.87±1.539	1.52±0.177	0.90±4.142
		R^2^	0.29636	0.34583	0.85317	0.33868
Power law 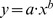		*a*	0.16±0.014	0.16±0.015	0.19±0.012	0.16±0.019
		*b*	−0.77±0.058	−0.78±0.060	−0.83±0.049	−0.76±0.081
		R^2^	0.74028	0.6772	0.86078	0.65361
Exponential 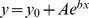		*y_0_*	0.00±0.002	0.00±0.002	0.00±0.002	0.00±0.003
		*A*	0.17±0.10	0.17±0.012	0.20±0.12	0.18±0.017
		*b*	−0.18±0.016	−0.18±0.017	−0.24±0.019	−0.19±0.026
		R^2^	0.89807	0.84248	0.93275	0.81939

R-squares (R^2^) in bold font and grey background represent the best fitted model for each network.

#### Degree correlation

**Figure 5:**
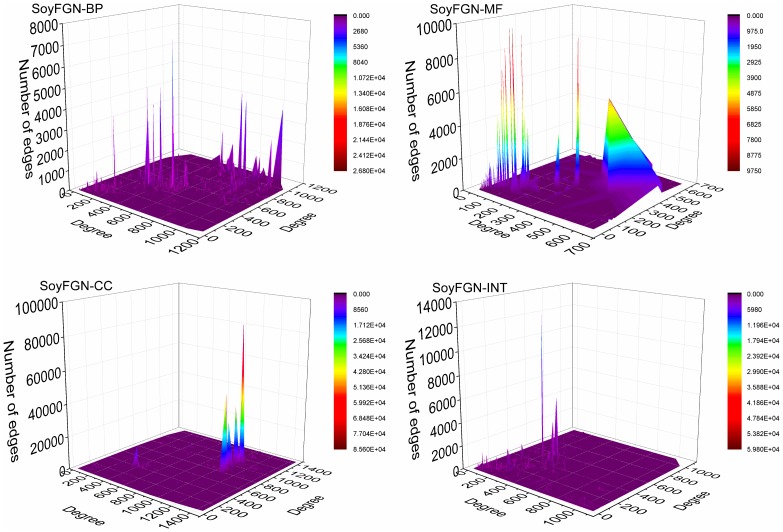
The joint degree distributions (JDD) of SoyFGNs. The X- and Y-axes represent the nodal degrees and Z-axis represents the number of edges of the pairwise degrees. The distributions show that genes in SoyFGNs tend to interact with the genes of the same degree, indicating the characteristic of assortativity.

The JDD of SoyFGNs in BP, MF, CC, and INT are visualised as a 3-D surface graph in Figure 5. The results suggest several important points. First, in all SoyFGNs, most of degree pairs have a small number of edges. The average numbers of edges are 38.66, 153.13, 88.46, and 26.15 for SoyFGN in BP, MF, CC, and INT, respectively, indicating the low network densities as shown in Table 2. Second, the extremely sharp protrusions show that little nodes share a large number of edges, indicating the existence of local dense functional modules in SoyFGNs. Third, the majority of apparent peaks (the number of edges ≥2000) appear in the low-low and high-high degree node pairs, suggesting that the genes tend to interact with those of similar degrees in SoyFGNs, indicating their assortative features.

**Figure 6:**
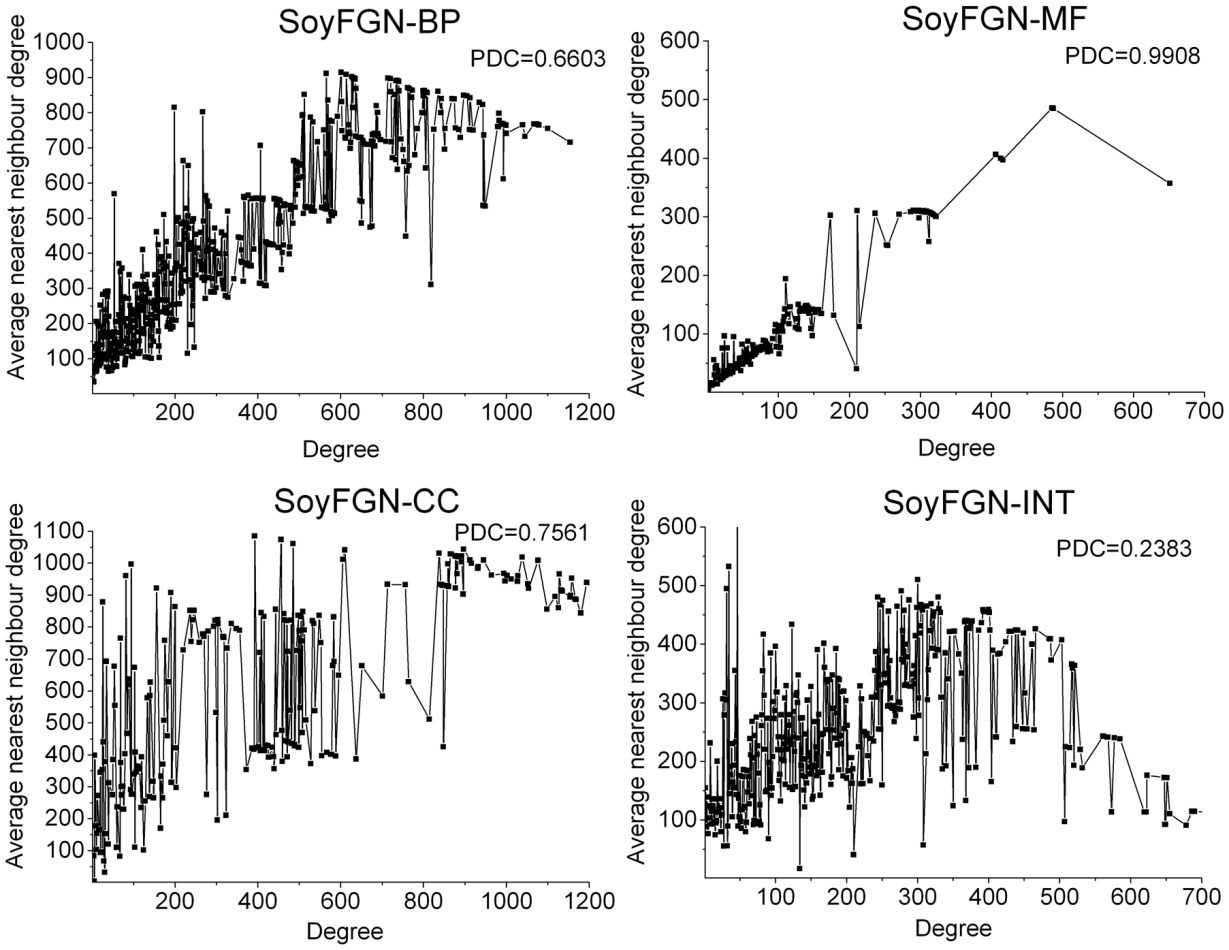
The k-nearest neighbours (*knn*) and Pearson degree correlations (PDCs) of SoyFGNs. Note that the PDC in each sub-graph was calculated according to the degree of two endpoints of all edges, rather than results derived from this graph.

Similar results were obtained in the analysis of the *knn* and the PDC (Figure 6). The overall ascending *knns* and large positive PDCs indicate that genes of similar degrees tend to be connected with each other more in all four SoyFGNs.

### SoyFGNs is more extensive and accurate than a network generated by orthology from Arabidopsis

**Figure 7:**
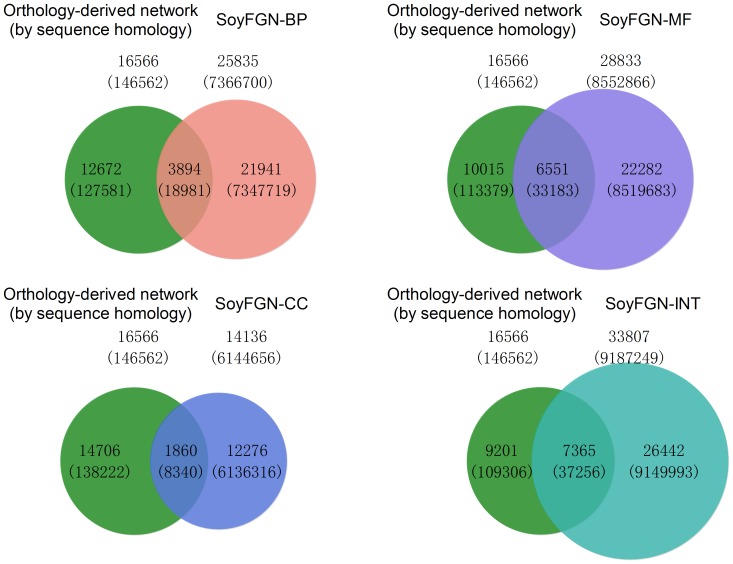
SoyFGNs include many genes and linkages beyond those found by simple orthology from the Arabidopsis gene network, as shown by four Venn diagrams of the gene linkages. The size of the pie corresponds to the number of edges. The numbers outside and inside the parentheses refer to the number of genes and the number of linkages in each network, respectively.

It is an open question how well a gene network derived from a better-characterised dicot such as Arabidopsis might faithfully reconstruct a gene network in another dicot such as soybean. To assess the accuracy of such a network, we defined an orthology-derived soybean gene network from Arabidopsis. The orthology-derived gene network covers 16566 soybean genes with 146562 links, whereas SoyFGNs cover 25835(9269 more genes), 28833(12267 more genes), 14136(2430 fewer genes) and 33807(17241 more genes) genes with 7366700, 8552866, 6144656 and 9187249 links in BP, MF, CC and INT, respectively (Figure 7). Therefore, in terms of genome coverage, SoyFGNs are more extensive than orthology-derived network. We further assessed the quality of SoyFGNs in comparison to orthology-derived network by two additional computational analyses using two independent data sources: KEGG pathways and co-expression profiles.

### Assessment using linkages derived from KEGG pathways

We tested the accuracy of the SoyFGNs versus the orthology-derived network using linkages derived from the KEGG pathway database. As a result, the KEGG-derived network consists of 380969 edges between 3144 genes, which share 238 KO IDs in 123 pathways of Soybean. The validation of a query network by the KEGG-derived network was based mainly on the gene coverage (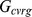) and the linkage accuracy (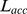) defined in Method section. Compared with orthology-derived network, SoyFGNs have significantly higher 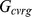 and 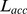 (Table 4), indicating SoyFGNs shared significantly more genes and linkages with KEGG-derived network than did the orthology-derived network. Assessed by linkages derived from KEGG pathways, SoyFGNs are therefore considered to be more extensive and accurate than orthology-derived network. We noted that SoyFGN-CC gets a lower 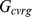 than orthology-derived network. This is mainly because (1) GO annotates the fewest number of genes in CC (see section 2.1.2) and (2) KEGG annotates genes mainly considering their molecular function (MF) or biological processes (BP) they participated while few about which cellular component (CC) they are.

**Table 4:** SoyFGNs are more extensive and accurate than orthology-derived network, validated by linkages derived from KEGG pathways.

Network	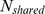 / 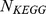	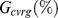	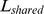 / 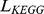	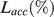
Orthology-derived	994/3144	31.62	219/37123	0.59
SoyFGN-BP	1791/3144	56.97	36785/147159	25.00
SoyFGN-MF	2110/3144	67.11	47583/198588	23.96
SoyFGN-CC	742/3144	23.60	6339/18506	34.24
SoyFGN-INT	2199/3144	69.94	57581/206116	27.94

### Assessment using linkages derived from co-expression profiles

We evaluated the SoyFGNs by comparing them to the orthology-derived network using the soybean gene co-expression network. As described in Introduction section, the co-expression network can, to some extent, reveal the genes function and the complex mechanism of action between genes, although genes interacting with each other do not always have similar gene expression profiles, and vice versa. Therefore we used the co-expression network as another independent reference network to evaluate the extent to which the more inclusive SoyFGNs consolidate the gene interactions derived from co-expression, referred to as inclusiveness. To do this, 7 of 11 in total Gene Expression Omnibus (GEO) [34] datasets were used to infer gene co-expression interactions, resulting in a co-expression network of 12933 genes linked by 2971228 edges. The inclusiveness of a network versus the co-expression network was also measured by the gene coverage (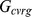) and the linkage accuracy (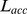), which led to similar results (Table 5), i.e., SoyFGNs shared more genes and linkages with the co-expression network than did the orthology-derived network, indicating the greater extensity and accuracy of SoyFGNs. Additionally, the statistics analysis showed that the shared edges (accuracy) are significantly higher than background (p<0.05, Table 5). Thus, according to the above two comparisons, reconstructing a gene network specifically for soybean genes rather than simply generating the network from orthology is essential and improves both accuracy and coverage of the network.

**Table 5:** SoyFGNs are more extensive and accurate than orthology-derived network, validated by linkages derived from co-expression profiles.

Network	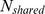 / 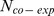	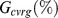	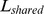 / 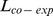	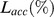	Average background 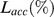	p-value
Orthology-derived	575/12933	4.44	472/23107	2.04	2.14	0.048144
SoyFGN-BP	5996/12933	46.36	24863/746387	3.33	0.59	0.008131
SoyFGN-MF	8235/12933	63.67	58727/1308942	4.49	0.46	0.004702
SoyFGN-CC	3286/12933	25.41	30725/239832	12.81	1.04	0.003726
SoyFGN-INT	9164/12933	70.86	91367/1496597	6.10	1.91	0.01437

p-value indicate the significance that the networks have a higher linkage accuracy than background.

### Network-guided discovery of disease-resistant genes

**Figure 8:**
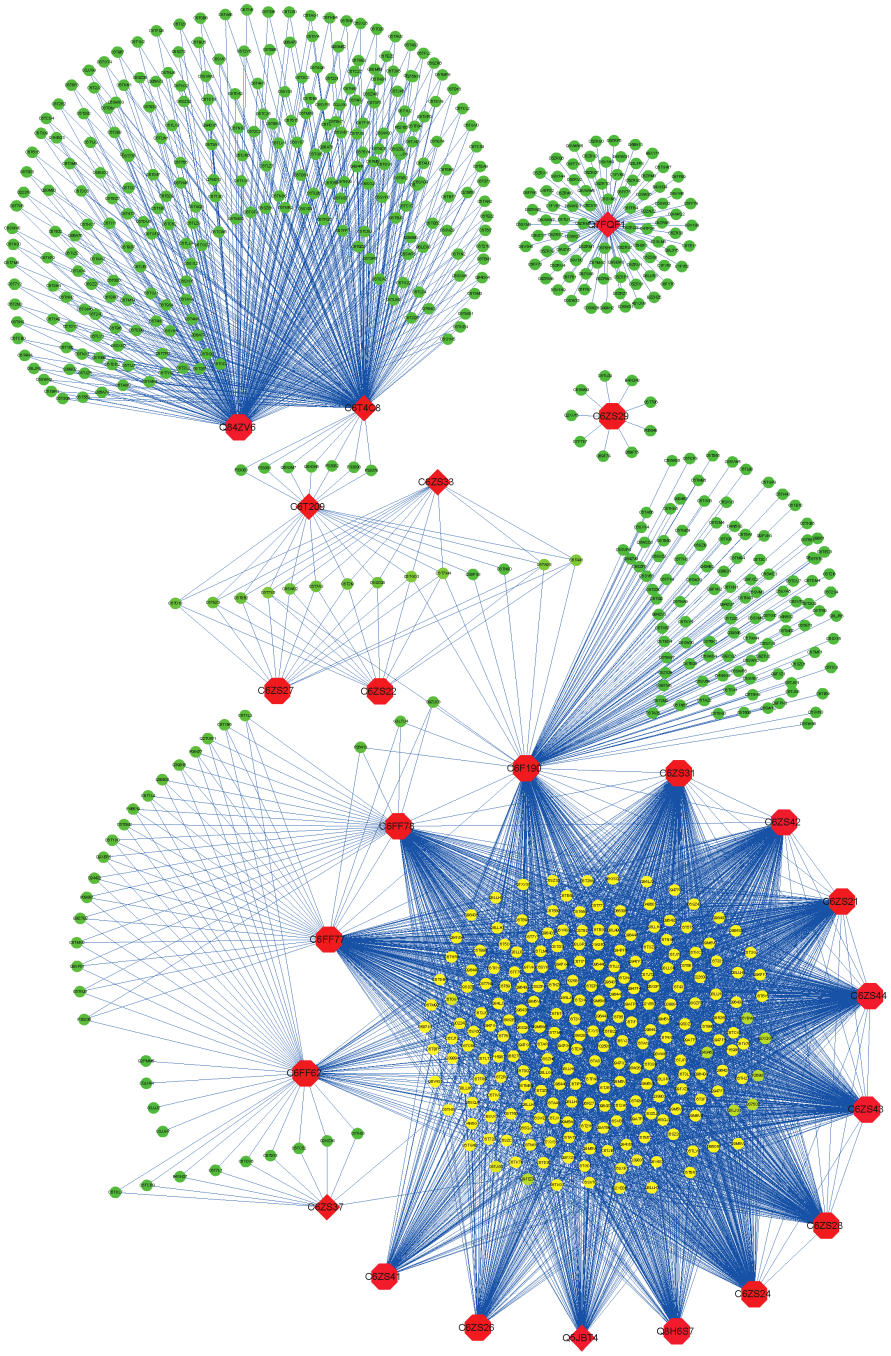
The 24 query genes (red filled) and the 737 candidate genes (ellipse). 6 putative R-genes are shaped like diamonds and 18 experimentally verified R-genes are shaped like octagons. The links between hunted candidate genes are not shown. A disease-resistant module is shown at the lower part of the figure, consisting of 225 candidates (yellow-filled ellipses) and surrounding 14 true R genes (red-filled octagons).

**Figure 9:**
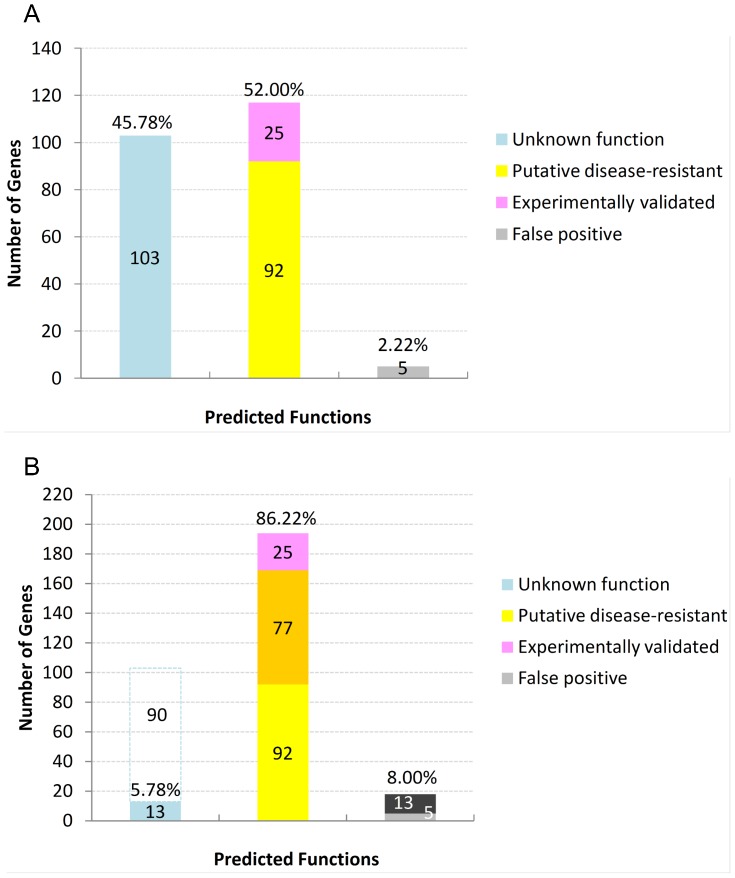
Network-guided discovery of disease-resistant genes. (A) Brief statistics of the 225 genes involved in the predicted disease-resistance module in SoyFGN-INT (before in silico verification). Numbers in the bars indicate the corresponding gene numbers. Percentages above the bars indicate the corresponding proportions. (B) The results after in silico verification, wherein the dark part of the same colour indicates the newly validated genes, which are those previous tagged with “Unknown function” in B (shown as the dashed box in C now). By in silico verification, 77 additional genes were predicted to be plant disease resistance genes.

Eighteen randomly selected true R-genes and six putative R-genes (listed in Table S2) were used as query genes to predict the potential R-genes in SoyFNG-INT by using guilt-by-association [36]. As a result, we identified 737 candidate genes with a predicted function in disease resistance, accounting for 95.0% of 776 genes deposited as disease resistance genes in UniprotKB as of June 2013. The 737 candidate genes and 24 query genes together constitute a disease-resistant gene network (Figure 8), which shows that most true R-genes (shaped like red octagons) are more connective with each other and share more candidates than the putative R-genes (red diamonds) do. We selected only the genes (14 genes, pink background in Table S2 or red-filled octagons in Figure 8) and their first neighbors (225 genes, yellow-filled ellipses in Figure 8) that constitute the largest disease-resistance module (listed in Table S3) as the high-confidence predictions to further evaluate the predictability of SoyFGN. Of these 225 disease-resistant candidates, 117 genes (52.00%) were previously known as R-genes, 25 of which were experimentally validated and 92 putative; 103 genes (45.78%), of which the functions were previously unknown, were newly predicted to be disease-resistant genes by using SoyFGN-INT; only 5 genes were not confirmed to be associated with plant disease resistance, i.e. false positive (2.22%). The results are also briefly summarised in Figure 9A.

### In silico verification of newly predicted disease-resistant genes

As the results of network-guided R-gene discovery, a highly confident disease-resistance module consisting of 14 query R-genes and 225 more predicted candidates was obtained by using the Gaussian smoothing guilt-by-association method. Among the 225 candidates (Table S3 (xls)), except 6 false-positive genes, 103 predicted candidates' functions are unknown. For these 103 newly predicted disease-resistant genes, it is nontrivial for us to validate them one by one using wet-lab experiments. Here we provide an *in silico* verification to check the performance of SoyFGN-INT on predicting the function of unknown genes by using Blast2GO [37]. The verification procedure includes the following: 1) BLAST the protein sequences of 103 candidates against the non-redundant protein database of NCBI (nr) using BLASTP to hunt for their orthologues; 2) analysis of the enrichment functions of BLAST hits by integrating the functional information retrieved from GO annotations, domain/motif and the KEGG pathways; 3) mapping the enrichment functions of orthologues to the unknown genes. We made many settings to reduce the false positive. In BLAST step, all sequences were blasted to nr (non-redundant protein database of NCBI) using BLASTP, with the minimum E-value of 1.0E-8; top 20 hits were selected to be used in next step; genes with less than 5 hits were excluded. In annotation step, in addition to GO annotations, we also ran the ‘InterProScan’ using all available applications as well as ‘GO-Slim’ using ‘goslim_plant.obo’ to enrich the annotations. Additionally, Enzyme codes and KEGG pathways were taken into account to enhance the annotations.

**Figure 10:**
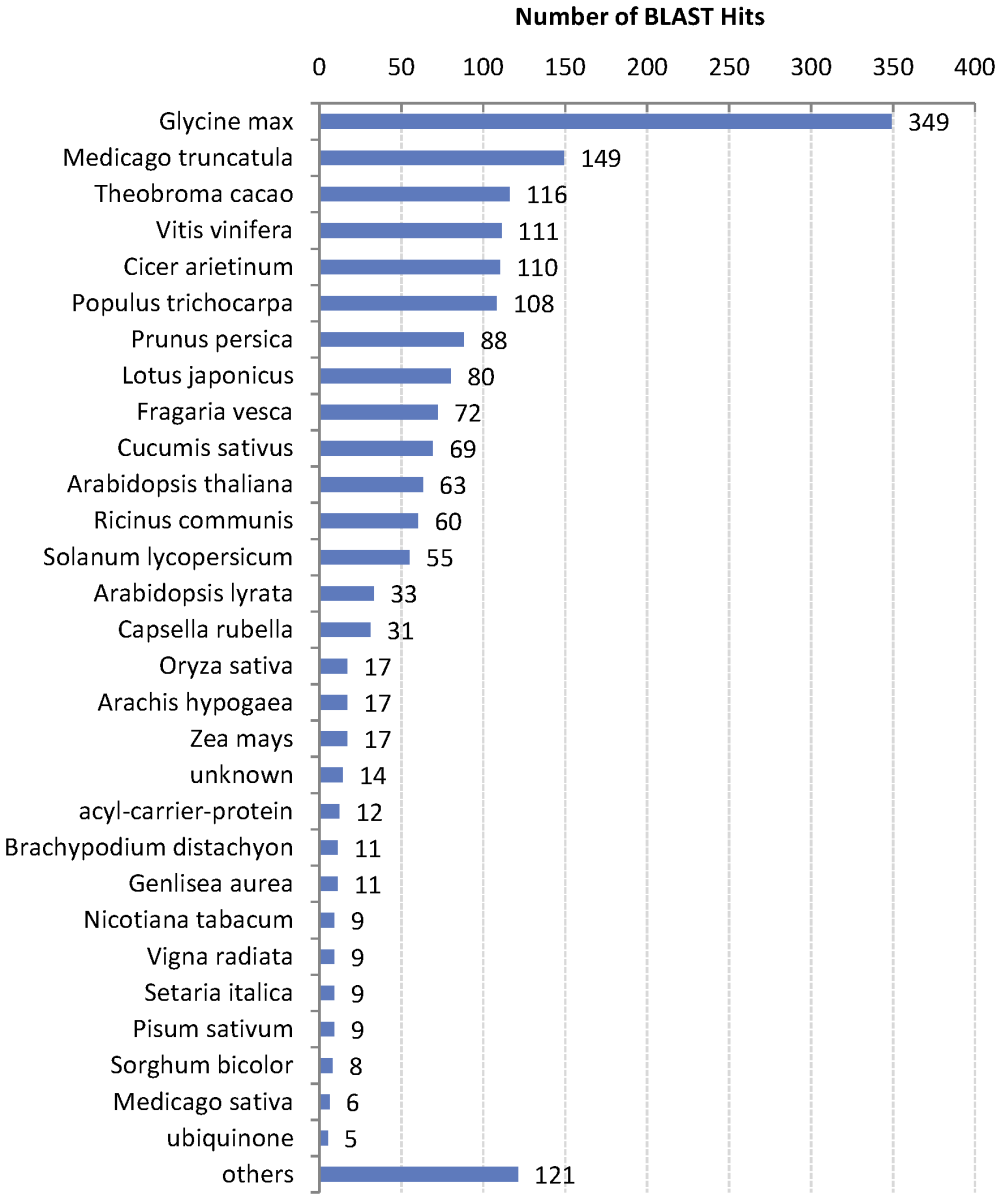
The species distribution of BLAST hits of the 103 genes.

**Figure 11:**
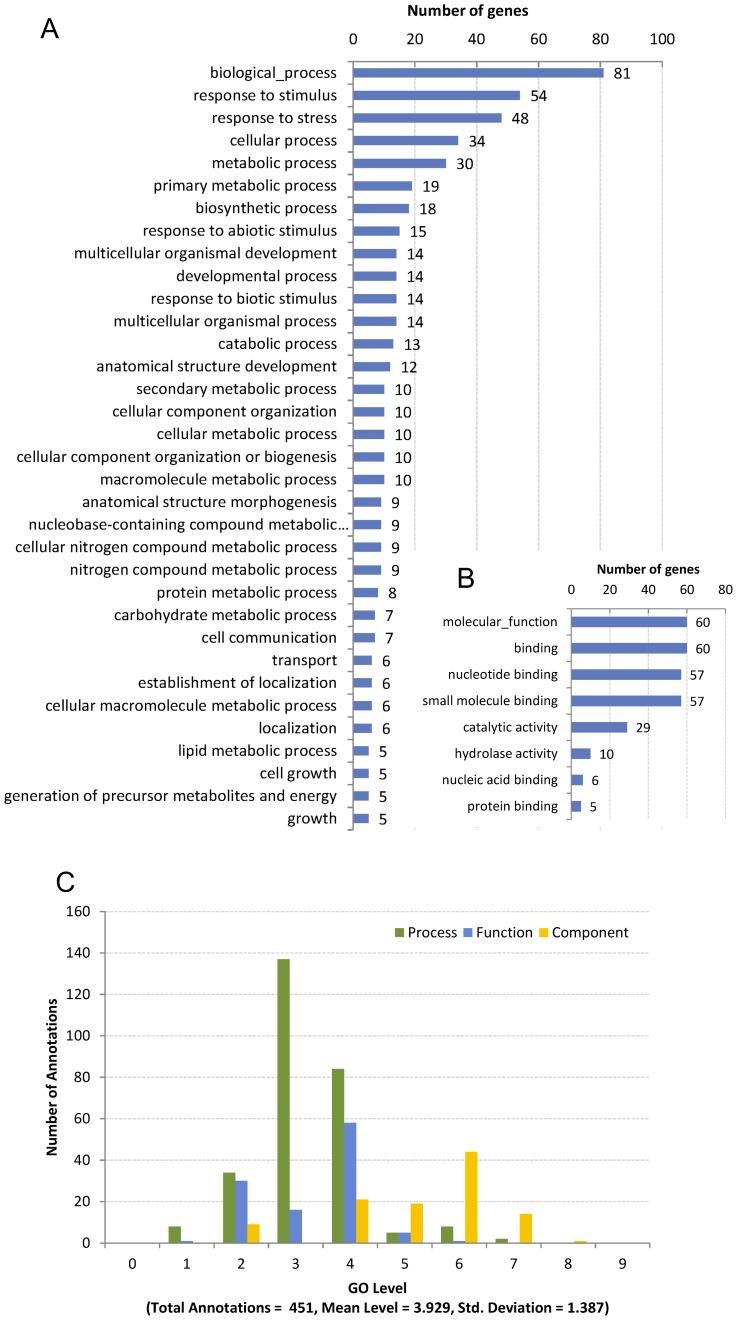
The enriched putative functions and GO-level distribution of 97 annotated genes. (A) The enriched functions in BP. (B) the enriched functions in MF. (C) The GO-level distribution. Only the functions, by which at least 5 genes were annotated, are shown.

As a results, 97 of the 103 genes got at least 1 hit, of which 89 genes got at least 10 hits and 87 genes got more than 20 hits (see Table S4). 97 matched genes finally got 451 annotations in three aspects of GO (P, biological process; F, molecular function; C, cellular component) in total (see Table S4). 14 matched genes were mapped to 8 Enzyme codes and involved in 18 KEGG pathways (Table 6). The species distribution of the annotations is shown in Figure 10. The enriched putative functions of all these 97 matched genes are shown in Figure 11. According to the annotations as well as the extensive database and literature searches, 77 of the 103 genes were newly predicted to be putative disease-resistant genes, 13 were recognised as non-resistant genes, and the rest (13 genes) remained unknown. Finally, 194 (86.22%) of 225 genes were identified as disease-resistant genes, an increase of 65.8% over the previously known R-genes, 13 retained unknown function and 18 were false positive (Figure 9B). Additionally, all predicted R genes were prioritised by assigning each with a weighted rating (WR) score. The results are provided in Table S4, which will help biologist identify the R-genes from the most likely candidates.

**Table 6:** 14 genes were mapped to 8 enzyme codes and involved in 18 KEGG pathways.

Pathway	#Genes in pathway	Enzyme	Enzyme ID	#Genes per Enzyme	Genes	Pathway ID
Oxidative phosphorylation	1	Dehydrogenase	ec:1.6.99.3	1	C6TJX1	map00190
Steroid degradation	1	dehydrogenase	ec:1.1.1.145	1	C6TFN3	map00984
Drug metabolism - cytochrome P450	1	dehydrogenase	ec:1.1.1.1	1	C6TH27	map00982
Arginine and proline metabolism	1	Cyclodeaminase	ec:4.3.1.12	1	C6TA12	map00330
Metabolism of xenobiotics by cytochrome P450	1	dehydrogenase	ec:1.1.1.1	1	C6TH27	map00980
Naphthalene degradation	1	dehydrogenase	ec:1.1.1.1	1	C6TH27	map00626
Thiamine metabolism	3	Phosphatase	ec:3.6.1.15	3	C6TAR2, C6T851, C6TIP6	map00730
Chloroalkane and chloroalkene degradation	1	dehydrogenase	ec:1.1.1.1	1	C6TH27	map00625
Steroid hormone biosynthesis	1	dehydrogenase	ec:1.1.1.145	1	C6TFN3	map00140
Purine metabolism	4	phosphatase	ec:3.6.1.15	3	C6TAR2, C6T851’6TIP6	map00230
Purine metabolism	4	Adenylpyrophosphatase	ec:3.6.1.3	1	C6T7M9	map00230
Glycine, serine and threonine metabolism	1	dehydrogenase	ec:1.1.1.1	1	C6TH27	map00260
Isoflavonoid biosynthesis	4	Reductase	ec:1.3.1.45	4	C6TD30, C6TLM0,C6TNS6, C6TB34	map00943
Flavonoid biosynthesis	1	4-reductase	ec:1.1.1.219	1	C6TFN3	map00941
Nitrogen metabolism	1	dehydrogenase	ec:1.6.99.3	1	C6TJX1	map00910
Glycolysis Gluconeogenesis	1	dehydrogenase	ec:1.1.1.1	1	C6TH27	map00010
Fatty acid degradation	1	dehydrogenase	ec:1.1.1.1	1	C6TH27	map00071
Tyrosine metabolism	1	dehydrogenase	ec:1.1.1.1	1	C6TH27	map00350
Retinol metabolism	1	dehydrogenase	ec:1.1.1.1	1	C6TH27	map00830

## Discussion

### System-level insight into the cellular interactome of non-model organisms becomes feasible

We have shown here that inferring, modelling, and analysing the intracellular interactome of a non-model species became a reality based on the notion of functional gene network (FGN). Although FGNs have been constructed for many model species, the methods cannot yet be extended to other infrequently studied species, such as *Glycine max*, due to the absence of sufficient heterogeneous and previously known omic-level interaction data as shown in Figure 1. Using GO annotations and our SSDD method, proposed for comparing gene functional similarity (FS), we identified the pairwise genes' FSs for soybean and further modelled the gene network on the notion of functional association. The schemes introduced here seem much simpler than those integrating heterogeneous omic data, yet it is currently the best solution for non-model species because the GO annotations actually provide a way to integrate diverse data into a single structured dataset. Inferring from orthologs, co-expression, and sharing KEGG terms are some alternative solutions, by which, however, the networks were proved to be less extensive and accurate than SoyFGNs. Additionally, as a case study, the successful application of SoyFGN-INT to predict the soybean disease-resistant genes further illustrates that SoyFGNs constructed on the basis of GO similarities can also provide system-level insight into the intracellular interactome as the networks of model organisms did, and this will speed up the discovery and definition of the function and interaction of genes that control important plant characteristics such as disease resistance, symbiotic nitrogen fixation, and protein and lipid synthesis in soybean. A study conducted on the soybean microRNA interactome based on SoyFGNs is an additional powerful evidence of the important roles of SoyFGN in future studies of the soybean functional interactome at the genome and microRNome levels[38].

### The first global view of soybean gene functional interaction

Soybean (*Glycine max*) is one of the most economically important crops and a major food source. A soybean whole-genome shotgun sequence of Glycine max var. Williams 82 was first reported in 2010 [16], which stimulated research on soybean at the genome level. The work introduced herein is the first study on soybean gene interaction on the genome level. We drew four functional gene networks (SoyFGNs), containing up to 70% of the soybean genes reported by EnsemblPlants (release 18, April 2013) and the construction of the second version SoyFGNs covering all genes is about to release. The topological analysis showed that, like other biological networks, SoyFGNs are scale free, and their degree distributions fit best to exponential and power-law distributions. Their degree correlations indicate that the genes of similar degrees tend to be connected with each other more in all four SoyFGNs, referred to as assortativity, implying the existence of functional modules in SoyFGNs. The achievements we report here will be fundamental to further studies on the interactome of soybean at the genome level. We admit that the SoyFGNs certainly contain false positives and even errors, just as or more than the model organisms contain. However, the effort involved in this work seems to be the best solution with the best outcome for such non-model organisms in absence sufficient data sources. The inherent deficiencies will certainly be overcome with increasingly enrichment of the data.

### Availability

Based on the research described herein, we developed a user-interactive web platform for information retrieval and analysis of the SoyFGNs and the aforementioned microRNA networks derived from SoyFGNs, SoyFN: http://nclab.hit.edu.cn/SoyFN/.

### Prospects

**Figure 12:**
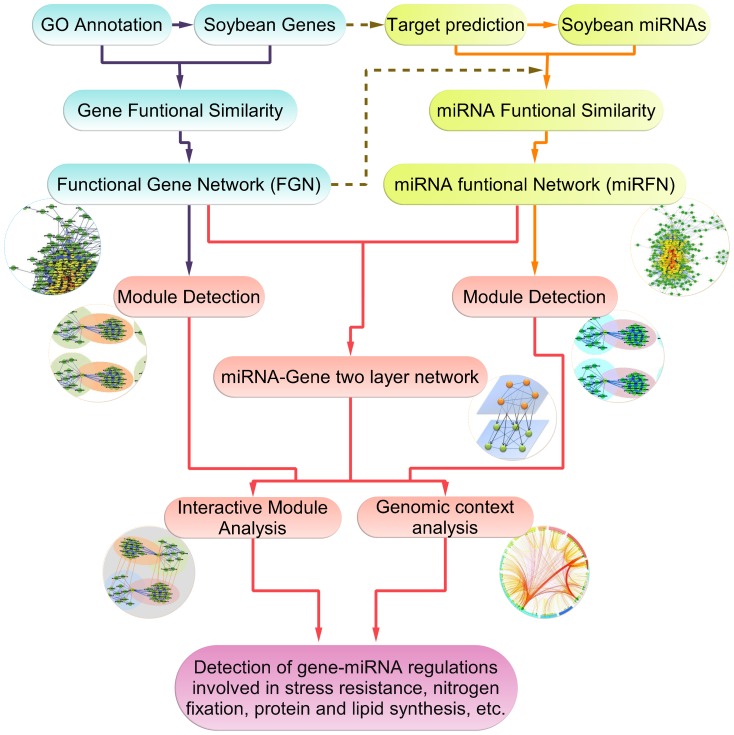
Schematic view of the work described herein as part of the whole research project for the soybean functional network. Rounded rectangles with blue backgrounds represent construction of soybean functional gene networks (SoyFGNs); yellow backgrounds represent construction of soybean miRNA functional networks (SoymiRFN) on the basis of SoyFGNs; red backgrounds represent our subsequent research prospects.

Our SoyFGNs provide a systematic view of the whole soybean genome, and hence such construction of the genome-wide networks has been followed by attempts to discover and predict function within the system as a whole. Therefore, the effort represented by our study is just the beginning of characterising the soybean functional genome. As shown in Figure 12, our whole research project consists of three main focuses: 1) construction of SoyFGN as described herein (shapes in blue background); 2) inferring the microRNA functional network of soybean based on the SoyFGNs (shapes in yellow background); 3) module detection, miRNA-gene two layer network analysis, and further interactive module analysis coupled with genomic context analysis to discover the gene-miRNA regulatory mechanism involved in stress resistance, nitrogen fixation, protein and lipid synthesis along with other biological processes in soybean (shapes in red background). Overall, the efforts of the study described herein are the basis of our further comprehensive studies on the soybean functional interactome at the genome and microRNome levels.

## Conclusions

As the most important biomolecules in a cell, genes rarely act alone. They interact functionally with other genes to synergistically mediate their biological functions. So far, hardly anything is known about this functionally interplay between genes in Soybean (*Glycine max*) at the genome level. The only large-scale genomic study in Soybean investigated the whole-genome shotgun sequences of Williams 82 [16], which stimulated our research on genome-level interactions among genes.

As an initial step on the way to fully expose the ensemble of all functional associations between genes, we here present the first FGNs of soybean (SoyFGNs). Instead of combining unavailable genomic, transcriptomic and comparative genomic data to predict associations (interactions) between gene pairs, we inferred the gene functional associations from GOA resulting in four comprehensive networks of gene associations that covers 70 percent of the predicted genes of soybean. We showed that SoyFGNs are scale free, and in which the genes of similar degrees tend to be connected with each other more in all four SoyFGNs, referred to as assortativity, implying the existence of functional modules in SoyFGNs. Verified by co-expression and KEGG pathways, SoyFGNs are more extensive and accurate than an orthology network derived from Arabidopsis. Network-guided disease-resistance gene discovery indicates that SoyFGNs constructed on the basis of GOA can also provide system-level insights into the intracellular interactome as the networks of model organisms did, which will speed up the discovery and definition of the function and interaction of genes that control important plant characteristics such as disease resistance, symbiotic nitrogen fixation, and protein and lipid synthesis in soybean. The availability of the predicted functional association network allows a gradual transition from a single gene perspective to a more comprehensive understanding of the complex biology of soybean. Additionally, a web tool for information retrieval and analysis of SoyFGNs can be accessed at SoyFN: http://nclab.hit.edu.cn/SoyFN.

## Supporting Information

Table S1 **Orthologs between soybean and Arabidopsis using BLASTN.**
(XLS)

Table S2 **Twenty-four query genes used in SoyFGN-INT-based prediction.**
(XLS)

Table S3 **The 225 candidate R-genes involved in the predicted disease-resistance module in SoyFGN-INT.**
(XLS)

Table S4 **The putative functional annotations of 103 unknown genes by using Blast2GO.**
(XLS)
